# Hardware-free MPFL reconstruction in patients with recurrent patellofemoral instability is safe and effective

**DOI:** 10.1186/s13018-022-03008-5

**Published:** 2022-02-22

**Authors:** Theodorakys Marín Fermín, Filippo Migliorini, Giorgos Kalifis, Bashir Ahmed Zikria, Pieter D’Hooghe, Khalid Al-Khelaifi, Emmanouil T. Papakostas, Nicola Maffulli

**Affiliations:** 1 Aspetar Orthopaedic and Sports Medicine Hospital, Doha, Qatar; 2 Department of Orthopaedic, Trauma, and Reconstructive Surgery, RWTH Aachen University Hospital, Pauwelsstr. 30, 52074 Aachen, Germany; 3 Department of Trauma and Orthopaedics, Hull Royal Infirmary, Hull, UK; 4 Department of Medicine, Surgery and Dentistry, University of Salerno, Baronissi, Italy; 5 School of Pharmacy and Bioengineering, Keele University School of Medicine, Stoke on Trent, England; 6 Centre for Sports and Exercise Medicine, Barts and the London School of Medicine and Dentistry, Mile End Hospital, Queen Mary University of London, London, England

**Keywords:** Hardware-free, Medial patellofemoral ligament, MPFL reconstruction, Patellofemoral instability

## Abstract

**Purpose:**

This systematic review evaluated the clinical outcomes of hardware-free MPFL reconstruction techniques in patients with recurrent patellofemoral instability, focusing on patient-reported outcome measures (PROMs), redislocation rate, and complications. The hypothesis was that hardware-free MPFL reconstruction in patients with recurrent patellofemoral instability is safe and effective.

**Methods:**

This systematic review was conducted following the PRISMA guidelines. PubMed, Scopus, and Virtual Health Library databases were accessed in October 2021. All the clinical studies investigating the efficacy and feasibility of hardware-free MPFL reconstruction were screened for inclusion. Only studies with a minimum 24-month follow-up were considered eligible. Kujala Anterior Knee Pain Scale improvement and redislocation rate after surgical treatment were evaluated as primary outcomes. The rate of postoperative complications was evaluated as a secondary outcome. The quality of the methodological assessment was assessed using the Modified Coleman Methodology Score.

**Results:**

Eight studies were included in the present systematic review. The quality of the methodological assessment was moderate. Short- to long-term improvement of Kujala score was observed in all included studies. Mean score improvement ranged from + 13.2/100 to + 54/100, with mean postoperative scores ranging from 82/100 to 94/100. Patellar redislocation was observed in 8.33% (8 of 96) patients.

**Conclusion:**

Hardware-free MPFL reconstruction with or without associated soft-tissue or bony realignment procedures provided reliable clinical improvements and was associated with a low rate of redislocation in patients with recurrent patellofemoral instability. Advantages such as safety, femoral physis preservation, and comparable complication profiles with implant-based techniques endorse its implementation. Orthopedic surgeons in cost-sensitive environments may also benefit their patients with lower costs, no need for implants, lack of implant-related complications, or surgery for implant removal.

*Level of evidence*: Level IV.

## Introduction

Patellar dislocation is the most common injury of the patellofemoral joint in young patients [1–3], with a high annual incidence at 147.7 per 100,000 in patients between 14 and 18 years, and recurrence rates reaching up to 70% after a primary dislocation [1, 2, 4–6]. Patients report giving away, joint effusion, anterior knee pain, limited range of motion, restricted sports activities participation and are at higher risk of developing osteoarthritis [2, 7]. The etiology of recurrent patellar dislocation is complex [1, 3, 8]. Trochlear or patellar dysplasia, patella alta, genu valgus or recurvatum, and increased femoral anteversion, and lateral tibial torsion have all been associated with an increased risk of patellofemoral dislocation [3, 9]. Therefore, several techniques, including proximal and distal realignment procedures, ligament reconstruction, or a combination of them, have been proposed for its management [2].

The medial patellofemoral ligament (MPFL) is the primary patellar restraint between 0° and 30° of knee flexion [3, 10–12]. Its anatomic reconstruction has shown satisfactory clinical outcomes, and it is considered a milestone in the management of recurrent patellofemoral instability [2, 3, 10, 13, 14]. Although many MPFL reconstruction techniques have been described, the ideal graft or fixation method are still debated [8, 15, 16]. Hardware-free fixation techniques, also called implantless, soft tissue, elastic, or dynamic fixation techniques, were initially developed to preserve the distal femoral physis in skeletally immature patients [17–21]. However, given their potential advantages, such as no implant-related costs, no need for hardware removal, and no implant-related complications, they are becoming increasingly popular [13, 22–28]. These advantages are particularly relevant in cost-sensitive populations [29, 30].

This systematic review evaluated the clinical outcomes of hardware-free MPFL reconstruction techniques in patients with recurrent patellofemoral instability. The focus was on patient-reported outcome measures (PROMs), redislocation rate, and complications. The hypothesis was that hardware-free MPFL reconstruction with or without associated soft-tissue or bony realignment procedures is safe and effective in patients with recurrent patellofemoral instability.

## Material and methods

### Search strategy

This systematic review was conducted following the Preferred Reporting Items for Systematic Reviews and Meta-Analyses (PRISMA) guidelines [31]. Two independent reviewers (TMF, GK) searched PubMed, Scopus, and Virtual Health Library databases in October 2021. The following terms, "medial patellofemoral ligament", "MPFL", "reconstruction", and "outcomes", were used alone and in combination with Boolean operators AND and OR. Inclusion and exclusion criteria were established before the search and were used to identify potentially eligible studies by title and abstract screening. Disagreements between reviewers were resolved by a third author (EP). The bibliographies of the included studies were also screened to identify additional studies.

### Eligibility criteria

All the clinical studies which investigated the efficacy and feasibility of hardware-free MPFL reconstruction were screened for inclusion. Given the linguistic abilities of the authors, only studies in English or Spanish were considered. Only studies with a minimum 24-month follow-up were considered eligible. Only studies that used the Kujala Anterior Knee Pain Scale as PROM. Reviews, commentaries, editorials, and opinions were excluded as were biomechanic and animal studies. Studies that did not properly describe the surgical procedure were also excluded. Missing data on the outcomes of interests warranted the exclusion from the present study.

### Data extraction

Two independent investigators (TMF, GK) reviewed the resulting articles and performed data extraction. For each included study, the following data were extracted: author, year, study design, patients demographic at baseline, length of the follow-up, surgical technique. Data concerning the Kujala Anterior Knee Pain Scale at baseline and at last follow-up were retrieved. The rate of complications was also collected.

### Outcomes of interest

The improvement in the Kujala Anterior Knee Pain Scale and redislocation rate after surgical treatment were evaluated as primary outcomes. The Kujala Anterior Knee Pain Scale is a 0–100 thirteen-question patient-reported outcome assessment tool widely used to evaluate the outcomes following surgical procedures in patients with patellofemoral instability [7]. A score of 95 points or greater was considered excellent, 94 to 85 as good, 84 to 65 as fair, and 64 or less as poor [32]. The rate of postoperative complications was evaluated as a secondary outcome.

### Methodological quality assessment

The quality of the methodological assessment was assessed using the Modified Coleman Methodology Score (mCMS) (Table 1) [38].

**Table 1:** Modified Coleman methodology scores of the included studies

Study	LOE	Type of study	Score
Abouelsoud et al. [27]	IV	CS	59
Lind et al. [18]	I	RCT	61
Maffulli et al. [33]	IV	CS	65
Malecki et al. [34]	IV	CS	60
Marot et al. [21]	IV	Multicenter longitudinal prospective comparative study	70
Monllau et al. [35]	IV	CS	70
Shimizu et al. [36]	IV	CS	57
Sobhy et al. [37]	IV	CS	61

CS, case series; LOE, level of evidence; RCT, randomized controlled trial

### Statistical analysis

The statistical analysis was performed using SPSS V.19 and Microsoft Excel 2016 (Microsoft®, USA). Continuous data were presented as mean values, standard deviations. Dichotomic data were presented as percentages. The t-test was used for continuous data, and the chi-square test for binary variables. *P* values < 0.05 were considered significant.

## Results

**Fig. 1:**
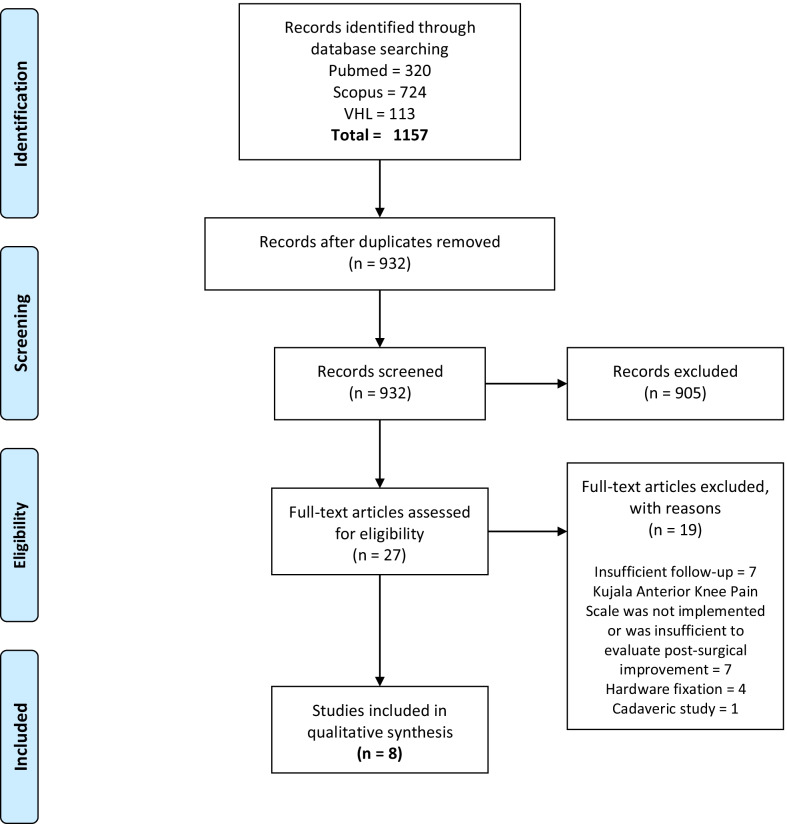
Flow-chart of the literature search

The initial literature search yielded 932 potentially relevant records after the removal of duplicates (*N* = 411). Titles and abstracts were independently screened, and 27 articles were selected for full-text evaluation. Seven studies were excluded because of insufficient follow-up [19, 39–44] and seven more because Kujala Anterior Knee Pain Scale was not used or data were insufficient to evaluate post-surgical improvement [45–51]. Finally, eight studies met the predetermined eligibility criteria, and no additional studies were included after citation screening in the systematic review (Fig. 1). There were six case series [27, 33–37], one multicenter longitudinal prospective comparative study [21], and one randomized controlled trial [18].

The descriptions of diagnosis and surgical techniques were consistent and accurate in most studies. The rehabilitation process was poorly described in some studies. All studies adequately reported outcome measures, the timing of outcome assessment, and the unbiased selection criteria of the subjects involved. Of the mCMS items, 'study size' and 'mean follow-up' scored the lowest because five out of eight studies had included less than 30 patients [18, 21, 27, 36, 37], and the follow-up was within 12–36 months in six of them [18, 21, 27, 33, 34, 37]. Furthermore, among these studies, six were case series [27, 33–37]. The lack of general health measures and the procedures for outcomes assessment were the most important limitations. It was unclear whether investigators were independent of surgeons, and completion of assessment by patients with minimal investigator assistance was not explicit in most studies. Recruitment rate was lower than 90% in five studies [18, 27, 33, 36, 37]. Concluding, the average mCMS value was 62.88 (range 57–70), demonstrating moderate methodological quality.

Narrative analysis of the collected data was conducted and summarized in Table 2.

**Table 2:** Outcomes of hardware-free medial patellofemoral ligament reconstruction techniques

Study	Number of patients	Patients' characteristics	Follow-up	Surgical technique	Outcomes	Complications
Abouelsoud et al. [27] Case series	16 patients	Age: 8–15 (11.5) years Sex: 5 males and 11 females Pediatric patients with recurrent atraumatic patellar dislocation and generalized ligament laxity No patient had TT-TG distance more than 20 mm, or severe trochlear dysplasia	24–34 (29.25) months	Single-bundle MPFL reconstruction using quadriceps tendon autograft Patellar fixation: the medial third of quadriceps tendon patellar attachment was preserved Femoral fixation: through stitches to the periosteum and bone in the MPFL femoral footprint and the adductor magnus tendon Graft tensioning: fixed at 30° of flexion before suturing Medial retinaculum plication was also performed	Kujala score: preoperatively 56 ± 4.72 points (range, 49–61) to postoperatively 94 ± 2.73 (range, 90–99), which is considered highly significant (*P* < 0.005) Mean Kujala score improvement: 38 No redislocation episodes were reported in any of the patients during the follow-up period	Flexion deficit: a patient (6.25%) reported losing the last 15° of flexion
Lind et al. [18] RCT	29 patients	Age: 18–46 (24.7) years Sex: 10 males and 19 females Patients with recurrent patellar instability with at least two lateral patellar dislocations and subjective instability symptoms with/without increased TT-TG distance or severe trochlear dysplasia	2 years	Double-bundle MPFL reconstruction using a gracilis tendon autograft Patellar fixation: two bone tunnels in the proximal half Femoral fixation: the graft was looped around the adductor magnus tendon as femoral fixation Graft tensioning: lightly tensioned at 30° of flexion before suturing 10 mm tibial tuberosity medialization was performed in 20% of patients, in which tibial tuberosity trochlear groove distance (> 15 mm for men and > 20 mm for women) was increased. Patients with chondral pathology were treated with debridement alone. No patients had cartilage repair procedures	Kujala score: 75.8 ± 11.9 preoperatively to 89 ± 10 with no difference between groups (*P* = 0.73) Mean Kujala score improvement: 13.2 However, the soft-tissue fixation group was lower in age, had a higher preoperative Kujala score, and had a lower proportion of severe trochlear dysplasia There were no redislocations in either of the 2 study groups	Pain at the medial femoral condyle: three patients (11%) in both study groups. No patients had moderate or severe tenderness on palpation at the patella Flexion deficit: one patient (3.33%) in the screw fixation group Subjective patellar instability: one patient (3.45%) in the soft-tissue fixation group
Maffulli et al. [33] Case series	34 patients	Age: 13–39 (26.5) years Sex: 7 males and 27 females Recreational athletes with chronic recurrent patellar dislocations. Patients had at least two documented patellar dislocations requiring reduction under sedation without malalignment or trochlear dysplasia No patient had received a previous MPFL reconstruction, but four (11.77%) of them had undergone other soft-tissue procedures. Thirteen patients (38.24%) had ICRS grade IV patellofemoral osteochondral defects	2–4.2 (3.5) years	Combined MPTL and MPFL reconstruction using a gracilis tendon autograft Tibial fixation: gracilis tendon distal attachment was preserved Patellar fixation: achieved through bone tunnels in the proximal and distal halves Femoral fixation: the graft was looped around the adductor magnus tendon Graft tensioning: established by manual 10 mm or one quadrant lateralization of the patella Osteochondral injuries were treated with debridement and/or microfractures	Kujala score: 47 ± 17 (range, 38–55) preoperatively to 82 ± 17 (range, 75–90; *P* = 0.02) postoperatively Mean Kujala score improvement: 35 No significant differences between patients with or without osteochondral lesions were found Three male patients (8.82%) had traumatic redislocation of the patella during sports activities	Drill-hole-related problems: 2 patients (5.88%) Hypoesthesia: 3 patients at 6-week follow-up. It persisted at final follow-up in one (2.94%) of them Anterior knee pain: 11 patients at 6-week follow-up persisted in 3 (8.82%) of them at final follow-up Osteoarthritis: 4 patients (11.76%) developed grade II osteoarthritis, and 3 (8.82%) developed grade III osteoarthritis
Malecki et al. [34] Case series	33 patients (39 knees)	Age: 8–18 (16) years Sex: 13 males and 20 females Patients with recurrent patellar dislocation Preoperatively, patellar tilt was observed in 25 knees and patellar shift in 32 knees. Twenty-one patients met the diagnostic criterion for ligamentous laxity (63.6%)	2–3 (2.6) years	MPFL reconstruction with adductor magnus tendon autograft Patellar fixation: sutured through a single bone tunnel Femoral fixation: adductor magnus tendon distal attachment was preserved Graft tensioning: at 30° of flexion In 9 knees with patellar shift and lateralization of the tibial tuberosity, concomitant Roux-Goldthwait partial patellar medial transposition was performed. In 23 knees with patellar tilt, lateral retinacular release was also performed. Distal realignment was done when the Q angle was greater than 20° and when an additional patellar shift and an increased congruence angle were present	Kujala score: 66 points (range, 38–88) preoperatively to 92 points (range, 70–100) postoperatively Mean Kujala score improvement: 26 Four patients (10.26%) presented patellar redislocation after surgery, three cases during sports activities and one case during dancing. The recurrent events occurred in patients without partial transposition of the patellar tendon	Positive apprehension test: 7 cases (17.9%) at final follow-up
Marot et al. [21] Multicenter longitudinal prospective comparative study	29 patients	Age: (22.8) years Sex: 11 males and 18 females Patients with objective recurrent (minimum two episodes of dislocation) patellar instability, without malalignment or severe trochlear dysplasia	2–5 years	Isolated quasi-anatomical double-bundle MPFL reconstruction using a minimum 180 mm length gracilis tendon autograft Patellar fixation: V-shaped tunnels in the proximal half Femoral fixation: the graft was looped around the adductor magnus tendon Graft tensioning: at 30° of flexion, allowing around 10 mm manual lateralization	Kujala score: 89.3 ± 8.5 postoperatively Mean Kujala score improvement: 27.3 ± 15.6 No statistical difference was found between the two groups Only one (3.45%) postoperative traumatic patellar dislocation occurred in the Isolated quasi-anatomical double-bundle MPFL reconstruction group at eight months postoperative during sports activities	Subjective patellar instability: two cases (3.51%) postoperatively, one in each group
Monllau et al. [35] Case series	35 patients (36 knees)	Age: (25.6 ± 9.4) years Sex: 17 males and 19 females Patients with objective recurrent patellar dislocations Twenty patients (55.6%) had increased TT-TG distance or patella alta	Minimum 27 (37.6) months	Quasi-anatomical double-bundle MPFL reconstruction with gracilis tendon autograft Patellar fixation: V-shaped bone tunnels in the superior third of the patellar medial border Femoral fixation: looped around adductor magnus tendon Graft tensioning: based on manual 10 mm lateralization at 30° of knee flexion An associated distal realignment procedure was performed in 20 patients (55.6%)	Kujala score: 63 (range, 49–70) preoperatively to 90 (range, 79–98) postoperatively (*P* < 0.001) Mean Kujala score improvement: 25 (range, 22–37) No patient experienced recurrent patellar dislocation in this series	Positive apprehension test: one patient (2.86%) Flexion deficit: two patients (5.8%), one of them required arthroscopic arthrolysis Hypertrophic wound scar: six knees (16.7%) No radiological progression of patellofemoral osteoarthritis was seen in any case at the final follow-up
Shimizu et al. [36] Case series	15 patients (20 knees)	Age: 11–41 (19.9) years Sex: 2 males and 13 females Patients with recurrent patellar dislocation Seven patients (35%) had patella alta, and six patients (30%) had osteochondral lesions	60–215 (123) months	Double-bundle MPFL reconstruction using a semitendinosus tendon autograft Patellar fixation: through a single bone tunnel in the patella, only one side of the tendon graft was passed, and the other side was sutured to it on the anterior patellar surface Femoral fixation: to femoral attachment of the medial collateral ligament through a 1 cm slit Graft tensioning: lateral patellar edge and lateral trochlear margin position maintained congruent and tensioned at 30° of flexion Additional Insall's proximal realignment procedure was done. Six knees (30%) with severe osteochondral patellar lesions were treated with osteochondral fixation (three knees) and osteochondral transplantation (three knees)	Kujala score: significantly improved from 65.5 ± 17.0 preoperatively to 86.7 ± 14.9 postoperatively (*P* < 0.05) Mean Kujala score improvement: 21.2 No redislocation was observed. One patient had a history of subluxation postoperatively	Positive apprehensive sign: five knees (25%) Limited range of motion: one (5%) at two months postoperatively and improved to a full range of motion after manipulation under anesthesia. No limited range of motion was observed at final follow-up in any patient Osteoarthritis: five knees (25%) had osteoarthritic change postoperatively. Four of these five knees had a severe osteochondral lesion preoperatively, and osteochondral fixation or osteochondral transplantation surgery had been performed simultaneously
Sobhy et al. [37] Case series	29 patients	Age: 17–26 (20.1) years Sex: 21 males and 8 females All patients included in our study had recurrent patellar dislocations, with normal patellofemoral bone morphology and limb alignment, with no other ligamentous deficiencies Four patients (13.8%) had a positive family history of frank patellar dislocation. Each patient had suffered at least two episodes of patellar dislocation. Two patients had previous arthroscopic lateral retinacular release, and two patients had previous ACL reconstruction Nineteen cases had a traumatic event, while 10 had no history of trauma	24–48 (32.2) months	Relay Technique: MPFL and TPFL reconstruction using semitendinosus tendon autograft Tibial fixation: semitendinosus distal attachment was preserved Patellar fixation: through bone tunnels Femoral fixation: achieved using a bone tunnel in the MPFL footprint. Graft and sutures were pulled in and tied in the opposite cortex Graft tensioning: tensioned in 20°–30° of flexion to approximately allow 5 mm of medial and lateral patellar glide	Kujala score: increased from 36.6 ± 6 (range, 22–48) preoperatively to 90.6 ± 7 (range, 78–100) postoperatively Kujala score values were significantly better in younger patients (*P* = 0.017) Mean Kujala score improvement: 54 No incidence of recurrence of patellar dislocation was detected in any case	Unstable feeling: 2 patients (6.9%). However, no positive apprehension or redislocation was found Flexion deficit: one patient (3.4%) reported a limited range of motion to 110° and inability to return to previous sports

ICRS, International Cartilage Regeneration and Joint Preservation Society classification, MPFL, medial patellofemoral ligament; MTFL, medial tibiofemoral ligament; RCT, randomized controlled trial; TT-TG, tibial tubercle-trochlear groove

Recurrent patellar dislocation was the main indication for hardware-free MPFL reconstruction in all the included studies [18, 21, 27, 33–37]. Three studies reported data from patients with physiological limb alignment and bone morphology [21, 33, 37]. Patients with increased tibial tubercle-trochlear groove (TT-TG) distance were included in three studies [18, 35], patella alta in two [35, 36], severe trochlear dysplasia in one [18], increased Q angle in one [34], concomitant general ligament laxity in two studies [27, 34]. Double bundle MPFL reconstruction using a free autograft [18, 21, 35, 36] was the most common technique, followed by single-bundle MPFL reconstruction with pedicled autograft [27, 34], and combined MPFL and MPTL reconstruction with pedicled autograft [33, 37]. Concomitant procedures included debridement [18, 33], microfractures [33], fixation of osteochondral lesions [36], osteochondral transplantation [36], distal realignment procedures [18, 34, 35], Insall's proximal realignment procedure [36], medial retinaculum plication [27], and lateral retinacular release [34].

The preferred method for patellar graft fixation was bone tunnels [18, 21, 33–37], except for Abouelsoud et al. [27] technique, in which the patellar tendon quadriceps attachment was preserved as a pedicled autograft. The most commonly used method for femoral fixation was looping the tendon graft around the adductor magnus tendon [18, 21, 33, 35]. Femoral fixation was also achieved by (1) suturing the graft to the periosteum and bone in the MPFL femoral footprint and the adductor magnus tendon [27], (2) a bone tunnel in the MPFL footprint [37], (3) looping it through a slit in the medial collateral ligament [36], and (4) preserving the adductor magnus tendon distal attachment when prepared as a pedicled autograft [34]. In combined MPFL and MPTL reconstruction, a gracilis tendon pedicled autograft was prepared to preserve its distal attachment [33, 37]. Graft tensioning and fixation at 30° of knee flexion was the favored method [18, 27, 34, 36], followed by 5–10 mm manual patellar lateralization [33], or a combination of both [21, 35, 37].

The gracilis tendon was the most commonly used autograft in the included studies [18, 21, 33, 35], followed by semitendinosus tendon [36, 37], quadriceps tendon [27], and adductor magnus tendon [34].

Short- to long-term improvement of Kujala score was observed in all included studies comprising patients from both sexes with mean ages ranging from 11.5 to 26.5 years [18, 21, 27, 33–37]. Mean score improvement ranged from + 13.2/100 to + 54/100, with mean postoperative scores ranging from 82/100 to 94/100. The final outcome was graded as good in seven studies [18, 21, 27, 34–37] and fair in one [33]. In two comparative studies, hardware-free MPFL reconstruction showed no statistical difference in Kujala score compared to femoral fixation using interference screws [18] or suture anchors [21]. Similarly, there were no statistical differences when comparing Kujala scores in patients with or without osteochondral injuries [33]. After surgery, patellar redislocation was observed in three of eight included studies [21, 33, 34]. Malecki et al. [34] reported four cases (10.26%), Maffulli et al. [33] three cases (8.82%), and Marot et al. [21] only one case (3.45%). All but one redislocations occurred during sports activities.

A positive apprehension test [34–37] and flexion deficit [27, 35–37] were the most commonly reported complications, ranging respectively from 2.86 to 25% and 3.4 to 6.25% overall. Other complications included osteoarthritis [33, 36], sensation of joint instability [18, 21], patella drill hole-related problems [33], hypoesthesia [33], anterior knee pain [33], pain at the medial femoral condyle [18], and hypertrophic wound scarring [35].

## Discussion

Hardware-free MPFL reconstruction with or without associated soft-tissue or bony realignment procedures provided short- to long-term improvement and a low redislocation rate in patients with recurrent patellofemoral instability, as initially hypothesized.

The number of bundles, type of fixation, and graft tensioning for MPFL reconstruction in patients with patellofemoral instability is still debated [2, 8, 10, 37, 52–58]. Thus, several variations and combinations of procedures have been described. Double-bundle MPFL reconstruction using a free gracilis autograft was the preferred method. Likewise, the most frequently implemented hardware-free fixation methods were patellar bone tunnels and looping the autograft around the adductor magnus tendon at 30° of knee flexion.

At least half of the world’s population lives in poverty and lacks access to quality essential health services [30]. Thus, investigations aiming to reduce the surgical-related burden represent a significant breakthrough for developing countries. Zhang et al. [59] exposed the contrasting cost differences of a pair of suture anchors and three high-strength sutures (US$800 vs. US$100, respectively) when comparing two different patellar fixation techniques. In fact, various authors have remarked on the high costs of suture anchors and interference screws [25, 26, 29]. Biomechanical studies have found no significant differences among fixation methods in MPFL reconstruction, and all provide higher failure loads than the native ligament [24, 60, 61]. Therefore, populations at economic disadvantage may benefit from hardware-free fixation techniques, being safe [18, 35, 37], cost-effective [3, 21, 35, 41, 42, 47, 59, 62]. Also, an effective hardware-free MPFL reconstruction can be performed in skeletally immature patients [3, 21, 27, 35, 36, 41, 42] and avoids implant-related complications or further surgery for implant removal [41, 42, 44].

Graft femoral fixation in hardware-free techniques is a debated technical point. Implant-based fixation techniques have shown similar pullout strength to hardware-free fixation techniques but higher stiffness [18, 60]. However, it has been suggested that the elastic behavior and lower stiffness of hardware-free fixation can result in a more compliant graft physiometry, lowering the risk of joint overconstrain and early-onset osteoarthritis [3, 18, 19, 21, 41, 46, 47]. Additionally, many hardware-free fixation techniques do not require intraoperative fluoroscopy, lowering associated costs and radiation exposure [21, 42]. It is still unknown whether higher fixation stiffness results in clinically relevant improvement or higher expenses.

In a recent systematic review, the clinical outcomes of patients with recurrent patellofemoral instability undergoing MPFL reconstruction using interference screws or anchors for autograft femoral fixation were compared [55]. The analysis of 19 clinical trials revealed no significant differences in Kujala Anterior Knee Pain Scale, Lysholm Knee Scoring Scale, and Tegner Activity Scale scores outcomes. The mean Kujala score improvement for anchor and interference screw fixation was 30.35 versus 35.75, respectively. The last follow-up scores were 86.23 ± 7.71 versus 88.37 ± 3.71 at a 46.5 ± 20.9 months follow-up, respectively. These results agree with the findings of the present systematic review and are further supported by additional studies which have not been included because, though published in peer-reviewed journals, they did not meet our strict inclusion criteria [19, 39–51]. On the other hand, the complication profile of hardware-free fixation shares similarities with implant-based fixation techniques, including subjective instability, positive apprehension test, and redislocation [55].

Hardware-free MPFL reconstruction was initially developed for skeletally immature patients to avoid growth damage to the distal femur physis [17, 51, 61, 63]. However, this technique has also been extended to the adult population [18, 33, 35, 36]. Indeed, among the studies considered in the present systematic review, only two included studies included solely patients younger than 18 years [23, 44], are adult population was the most commonly investigated [18, 21, 33, 35–37]. These findings confirmed a trend towards hardware-free techniques implementation regardless of the patient's age.

The present study certainly has some limitations. Only three studies reported information on isolated hardware-free MPFL reconstruction. Five studies combined MPFL reconstruction with additional soft-tissue or bony realignment procedures [18, 27, 34–36], and three studies with other treatments addressing to osteochondral injuries [18, 33, 36]. The combination of such procedures limits the extent of the findings of the present systematic review. Nevertheless, more than two-thirds of the patients presenting recurrent patellar dislocations demonstrate two or more pathoanatomical predisposing factors, which may synergistically predispose them to joint instability [64–66]. The association of additional procedures is still debated and should be evaluated at an individual level [1, 67]. Six of eight studies were case series, thus negatively impacting the overall quality of the results. Future comparative studies should follow a cost-effectiveness analysis methodology to find the most efficient MPFL reconstruction technique. The clinical relevance of the present systematic review is that the use of hardware-free MPFL reconstruction fixation techniques may represent an effective alternative for the surgical treatment of recurrent patellofemoral instability in cost-sensitive environments. Orthopaedic surgeons may benefit their patients with lower costs, no need for implants, lack of implant-related complications, and further surgery for implant removal.

## Conclusion

Hardware-free MPFL reconstruction provided clinical improvement and was associated with a low redislocation rate in patients with recurrent patellofemoral instability. Advantages such as safety, femoral physis preservation, and comparable complication profiles with implant-based techniques endorse their implementation. Orthopaedic surgeons in cost-sensitive environments may also benefit their patients with lower costs, no need for implants, lack of implant-related complications, and surgery for implant removal.

## Data Availability

This study does not contain any third material.

## Abbreviations

mCMS: modified Coleman Methodology Score
MPFL: Medial patellofemoral ligament
MTFL: Medial tibiofemoral ligament
PRISMA: Preferred Reporting Items for Systematic Reviews and Meta-Analyses
PROMs: Patient-reported outcome measures
TT-TG: Tibial tubercle-trochlear groove
